# Posterior Basal and Epidemic Cerebro-Spinal Meningitis

**Published:** 1908-03-07

**Authors:** 


### POSTERIOR BASAL AND EPIDEMIC CEREBRO-SPINAL MENINGITIS.

Posterior basal and epidemic cerebro-spinal meningitis can to some extent be diagnosed by the clinical picture presented by the patient, more especially by the very extreme retraction of the head in some cases, the "pyrexial crises" in others, and by the existence of the well-known skin eruptions in a few. Upon the whole, however, it may almost be said that the one clinical point that distinguishes posterior basal from tuberculous meningitis is the course of the two diseases. If the illness lasts about three weeks and ends in death it is extremely difficult to say for certain that the meningitis is not tuberculous. If, on the other hand, the patient lingers on for eight or ten weeks, the diagnosis of posterior basal meningitis is more likely to be right than that of tuberculous would be, even if the patient dies. If the patient, having been ill either three weeks or longer, ultimately gets well, the probability is that the trouble was meningococcal.

But it is unsatisfactory to have to await events before being able to say anything definite to the patient's friends. It is always well to be able to give a definite diagnosis at the earliest possible moment. Hence the value of laboratory help in these cases.

The best-known laboratory method of arriving at a diagnosis of meningococcal affection of the meninges is by making cultivations from the patient's cerebro-spinal fluid. The objection to it is that lumbar puncture is necessary before this fluid can be obtained. It is the main purpose of this article to show that there are means of diagnosing the disease without necessity for lumbar puncture.

Apart from the question of how much damage may be done to the nerves of the cauda equina, the operation is one to which the patient's friends do not readily consent. It is, of course, a fairly certain method of diagnosis if the fresh cerebro-spinal fluid, collected in a sterile tube, is forthwith sent to a bacterioligical laboratory. It is there cultivated, and the growth that results is examined microscopically, the report coming back to say whether the meningococcus is present or not.

The same end can, however, be attained by the much simpler procedure of pricking the patient's ear, collecting a few drops of blood in Widal's pipette, sealing the ends, and sending it to the laboratory. The two procedures there carried out are: (1) Determination of the agglutinative power of the serum for meningococci; and (2) determination of the opsonic index of the serum to meningococci.

Agglutination, or the "clumping reaction," is well known in connection with typhoid fever. It is, perhaps, less recognised that it is a phenomenon by no means restricted to typhoid bacilli. It seems that patients suffering from various bacterial diseases produce in their blood certain substances, known as "agglutinins," which act upon the specific microbes of the disease in question and cause them to "clump." Moreover, the agglutinating reaction is specific. That is to say, the serum of a patient suffering from typhoid fever contains agglutinins which cause typhoid bacilli to clump, but which do not cause other organisms to clump. So, too, the serum of patients suffering from bacterial dysentery causes Shiga's bacillus, but not other organisms, to clump. There is a specific clumping reaction for the micrococcus melitensis in Malta fever cases, and so on. There is a very mild degree of the same kind of reaction in regard to the meningococcus when it is mixed with blood serum from patients suffering from meningococcal disease.

The extent of the reaction is this: that whereas emulsions of meningococci, mixed with ordinary undiluted blood serum, do not clump at all, the same emulsions mixed with thrice-diluted serum from patients with epidemic cerebro-spinal meningitis will often clump within a quarter of an hour. Absence of clumping does not exclude the disease; but a positive reaction is considerably in favour of its presence.

Opsonic index estimations, performed with blood serum in the now familiar way, are very valuable in determining whether a patient's illness is due to the meningococcus or not. There is no need to enter upon the details of the laboratory procedure here. All that need be sent there is a little serum. The opsonic power of normal serum against the meningococcus is very slight; that is to say, the leucocytes take up very few of these organisms when they are incubated in normal serum. Quite the reverse is true of the serum of patients suffering from the particular form of meningitis that is under discussion. In many cases the leucocytes are so crowded with the cocci that the latter are difficult to count. The opsonic index of the serum of such patients to meningococci is often four, five, or more times greater than that of other persons.

Drs. Houston and Rankin, who worked much at the subject during the Belfast epidemic of cerebro-spinal meningitis, came to the conclusions:—

1. That the opsonic index in every case but one examined by them was over four from the sixth day of the disease onwards; in many cases it was already as high as this on the second day.

2. That the clumping reaction referred to above was never observed until the opsonic index had reached five times the normal; that is to say, that the opsonic index method is a much more delicate and early test for detecting meningococcal infections than is the clumping reaction.

3. That the combination of the clumping reaction and the opsonic index estimation together constitute as sure a method of diagnosing between meningococcal and other forms of meningitis as we have at our disposal.

It must be mentioned that, just as Calmette's tuberculo-ophthalmic reaction is absent in very acute and severe cases of tuberculosis, so there is neither clumping reaction nor increase of the opsonic index to meningococci in very rapid cases of epidemic cerebro-spinal meningitis. The majority of cases, however, are subacute rather than fulminating, and in them, especially from the sixth day of the illness onwards, the taking of a little blood and testing it for clumping and for the opsonic index to meningococci is quite as great a help in diagnosis as is cultivation of the cerebro-spinal fluid after lumbar puncture.

